# Anti‐anemic potential of *Eruca sativa* L. in iron‐deficient rat model; network pharmacology profiling

**DOI:** 10.1002/fsn3.4314

**Published:** 2024-07-21

**Authors:** Sana Javed, Zainab Shahzadi, Zubaida Yousaf, Irfan Anjum, Arusa Aftab, Samina Hanif, Zainab Maqbool, Riaz Ullah, Muhammad Ahmer Raza, Zafar Iqbal

**Affiliations:** ^1^ Department of Botany Lahore College for Women University Lahore Pakistan; ^2^ Department of Basic Medical Sciences Shifa College of Pharmaceutical Sciences, Shifa Tameer‐e‐Millat University Islamabad Pakistan; ^3^ Department of Pharmacognosy College of Pharmacy King Saud University Riyadh Saudi Arabia; ^4^ Department of Social and Clinical Pharmacy, Faculty of Pharmacy in Hradec Králové Charles University in Prague Prague Czech Republic; ^5^ Department of Surgery College of Medicine, King Saud University Riyadh Kingdom of Saudi Arabia

**Keywords:** active compounds, GC–MS, in vivo activity, iron deficiency anemia, network pharmacology

## Abstract

Iron deficiency anemia is a global health concern, affecting around 2 billion people. Oral iron therapy often causes severe gastro‐intestinal issues. *Eruca sativa*, member of the Brassicaceae family, is valued in traditional medicine and renowned for its rich iron and vitamin C content. This study aims to evaluate the anti‐anemic properties of *E. sativa* extract in vivo and identify its compounds targeting anemia mechanisms using network pharmacology. Thirty‐two Sprague–Dawley rats (200 ± 250 g) were split into two distinct groups, iron‐deficient and iron‐sufficient. Three different doses (200, 400, and 800 mg/kg) of aqueous extract of *E. sativa* were checked against anemia by studying hematological, oxidative stress, and histopathological parameters. GC–MS analysis of *E. sativa* revealed its phytochemical profile, followed by ADME screening. Network pharmacology explored targets related to iron deficiency anemia, with oral bioavailability and drug likeness assessment for compounds. The administration of extracts significantly improved various blood parameters, including osmotic fragility, Hb, RBCs, MCV, PCV, and alkaline phosphatase; catalase activity; and histopathological parameters such as liver in both iron‐deficient and iron‐sufficient rats (*p* < .001). Seventy‐nine compounds were identified in *E. sativa* aqueous extract, with only six of them found to be bioavailable and drug‐like against multiple targets. Gene ontology and pathway analysis revealed their diverse molecular, biological, and cellular functions. One gene EGFR was found to have functional association with ID anemia, suggesting potential for using *E. sativa* extracts. The study concludes that *E. sativa* extract has potential for iron deficiency anemia treatment, offering hope for future pharmaceutical interventions.

## Graphical abstract

The anti‐anemic potential of *Eruca sativa* L. has been validated in iron‐deficient rats. The hematological, oxidative stress, and histopathological parameters presented the effectiveness of *Eruca sativa* in anemic rats. ADME studies supported the biochemical findings. Key Findings: (1) The administration of *Eruca sativa* significantly improved blood profile, antioxidant enzymes, and liver function tests. (2) Gene ontology presented EGFR to have functional association with anemia, suggesting potential for using *E. sativa* extracts. (3*) E. sativa* extract has potential for iron deficiency anemia treatment.

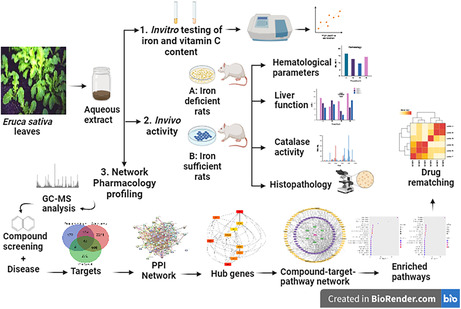

## 1 INTRODUCTION

Iron is a trace metal that is involved in many processes of life, including the transport of oxygen, redox processes, and the production of nucleic acids. Iron deficiency and iron deficiency anemia are global health problems that are faced by developed and developing countries (Pivina et al., 2019). Around 2 billion people worldwide are affected by iron deficiency (Lanzkowsky, 2016). The incidence of anemia has been as high as 35%–75% in developing countries compared with only 19% in developed countries (Lanzkowsky, 2016). A report by Aga Khan University quoted statistics showing a prevalence of 45% iron deficiency anemia in Pakistan. Studies have shown that anemia affects 41.7% to 77.0% of women in Pakistan (Ali et al., 2020). Anemia is characterized by low erythrocyte count or hemoglobin levels below recommended levels, while iron deficiency can occur without anemia and is indicated by low plasma ferritin and transferrin saturation (Özdemir, 2015). A wide spectrum of patients receives frequent prescriptions for iron supplements to prevent and cure iron deficiency (ID) and iron deficiency anemia (IDA); however, oral iron frequently results in serious gastrointestinal side effects such as constipation, stomach pain, nausea, and bloating (Grzywacz et al., 2017). Herbal remedies have the potential to be additional or alternative treatments for iron deficiency anemia that are risk‐free, do not affect the digestive system, and have fewer side effects (Fitriani et al., 2020).

*Eruca sativa* belongs to the family Brassicaceae, which is a good source of micro‐ and macronutrients. Family Brassicaceae is crucial for minerals like magnesium, iron, and calcium as well as vitamins A, B12, B6, C, E, and K (Raza et al., 2020). *E. sativa* dried leaves had high levels of iron and zinc apart from the oxalate content (Keyata et al., 2020). Mg, Ca, Fe, and K are the most common minerals in leaves. Its medicinal benefits as an astringent, diuretic, digestive, tonic, laxative, and stimulant are widely known in traditional medicine (Sharma et al., 2012). This herb also promotes hair growth and possesses antibacterial, antidiabetic, antihypertensive, antiplatelet, and antioxidant properties (Jaafar & Jaafar, 2019). Various in vivo studies proved that the plant extracts are used for the treatment of iron deficiency anemia. Aqueous extract of *Moringa indica* improved iron deficiency anemia and also enhanced disaccharides activity (Modupe & Oladiji, 2016). According to (Oladiji et al., 2007), in vivo studies of aqueous extract of *Sorghum bicolor* improve hemoglobin level and cure iron deficiency anemia. Administration of aqueous extract of *Panax japonicas* enhances blood deficiency in anemic rats (Zhang et al., 2014). In another study, Liu et al. (2021) reported that decoction of *Danggui Buxue* is effective for anemia. Furthermore, it was proved through network pharmacology and molecular docking that this plant has certain compounds that affect target genes for the cure of anemia.

Network pharmacology is essential in terms of efficacy, mechanism of action, systematic design of compatibility, drug development, and safety (Li et al., 2019). Nowadays, more and more complicated diseases are being investigated by merging multi‐omics technology with computer science in order to record the monolithic physiological response of humans (Singh et al., 2019). The analysis of big data in network pharmacology enables the identification of the synergistic effects of multi‐molecule medications, thus establishing a practical foundation and effective approach for the exploration and advancement of traditional Chinese medicine (TCM) (Liu et al., 2022). As *E. sativa* plant has a potential therapeutic effect against iron deficiency anemia due to high content of iron, vitamin C, and active phyto‐constituents. But this plant is poorly explored for anti‐anemic properties.

The present study was designed to investigate the anti‐anemic effects of *E. sativa* and the interaction of active compounds against anti‐anemia targets through network pharmacology profiling. This study also describes the ability of plant extract to interact with multiple targets, which can enhance its therapeutic efficacy. The main objective of the study was to use plant for iron deficiency anemia that is not only a rich source of iron but also helps in its maximum absorption with better laxative properties as compared with other allopathic medicines.

## 2 MATERIALS AND METHODS

### 2.1 Collection of plant material

Leaves of *Raphanus sativus* L., *Brassica campestris* L., *Brassica rapa* L., and *Eruca sativa L*. were collected from cultivated areas of Shakargarh. Voucher specimens were authenticated and submitted at the Prem Madhan Herbarium of the Botany Department of LCWU, Lahore with accession no *R. sativus* (LCWU‐0246), *B. campestris* (LCWU‐0695), *B. rapa* (LCWU‐0697), and *E. sativa* (LCWU‐1019).

### 2.2 In vitro activity

#### 2.2.1 Determination of iron in selected plants spp.

The iron content of all selected plants was determined by using a UV visible spectrophotometer (UV‐6000) according to the methodology of Thangiah et al. (2022).

#### 2.2.2 Determination of vitamin C content in selected plant samples

The ascorbic acid of all selected plants was determined by using a UV–visible spectrophotometer, following the methodology of Elgailani et al. (2017).

### 2.3 In vivo activity

Following in vitro testing, the plant which showed high iron and vitamin C content was selected for further investigation in in vivo studies. Among all selected plant spp., *E. sativa* L. showed high iron and vitamin C.

#### 2.3.1 Sample preparation

The dried plant sample (600 g) of *E. sativa* was dissolved in distilled water by 1/10 (W/V) and thoroughly mixed for 2  h with the aid of a magnetic stirrer. The solution was filtered using Whatman filter paper (0.10 m). Then, distilled water was evaporated from the filtrate with the help of a rotary evaporator. After the filtrate was concentrated in the water bath at 40°C. The remaining crude extract was reconstituted in distilled water to form doses of 200 mg/kg and larger doses of 400 and 800 mg/kg. The reconstituted aqueous extract was given orally to each animal in each group according to Oladiji et al. (2007).

#### 2.3.2 Animal model and laboratory conditions

This study included 32 rats (Sprague–Dawley, 200 ± 10 g), which were purchased from the “Animal House” of the Faculty of Pharmacy, The University of Lahore, Lahore (Ref. no. Bot/LCWU/1521‐A). Rats were kept for 12 h in the light/dark cycle along with the free approach to food and water with sustained temperature (21 ± 3°C), according to guidelines of European Community Guidelines. This study was conducted with the approval of the institutional research Ethics Committee of the Faculty of Pharmacy, The University of Lahore, and research Ethical Committee of Lahore College for Women University, Lahore, Pakistan (Ref. no. Bot/Lcwu/1521‐A).

#### 2.3.3 Feed composition and anemia induction

Iron‐deficient (ID) and iron‐sufficient (IS) diets were formulated with slight modifications from the components according to the methodology of Modupe and Oladiji (2016). Animals were equally divided into two groups: A and B. Group A was supplied with the iron‐deficient (ID) diet and group B was supplied with an iron‐sufficient diet (IS) for 6 weeks respectively. The feed component of iron‐deficient and iron‐sufficient diet is mentioned in Table 1.

**TABLE 1:** Feed components of ID and IS diet.

Feed components	Iron‐deficient diet (g/kg)	Iron‐sufficient diet (g/kg)
Bean seeds	550	550
Corn starch	40	40
Soybean oil	40	40
Sucrose	100	100
Methionine	15	15
Lysine	10	10
Vitamin mix	25	25
FeSO_4_.7H_2_O	–	35.06

Soybean oil: polyunsaturated fatty acids (58%), monounsaturated fatty acids (29%), saturated fatty acids (13%). (Vitamin mix (per kg of diet): vitamin A, 100,000 lU; vitamin D3, 10,000 lU; vitamin E, 100 mg; vitamin B1, 20 mg; vitamin B2, 40 mg; d‐calcium pantothenate, 100 mg; vitamin B6, 15 mg; vitamin C, 250 mg; vitamin K3, 15 mg; folic acid, 5000 mcg; nicotinic acid, 200 mg; biotin, 150 mcg; inositol, 80 mg).

#### 2.3.4 Study design

After 6 weeks, rats of Group A‐ID and Group B‐IS were divided into 4 groups with an equal no of rats (*n* = 4) in each group.

- A1: Iron‐deficient rats were given 200 mg/kg of aqueous extract of *E. sativa* L. for 7 days.
- A2: Iron‐deficient rats were given 400 mg/kg of aqueous extract of *E. sativa* L. for 7 days.
- A3: Iron‐deficient rats were given 800 mg/kg of aqueous extract of *E. sativa* L. for 7 days.
- A4: Iron‐deficient rats orally administered daily for 7 days with 1 mL of the vehicle (distilled water) designated as ID‐control.
- B1: Iron‐sufficient rats were given 200 mg/kg of aqueous extract of *E. sativa* L. for 7 days.
- B2: Iron‐sufficient rats were given 400 mg/kg of aqueous extract of *E. sativa* L. for 7 days.
- B3: Iron‐sufficient rats were orally administered 800 mg/kg of aqueous extract of *E. sativa* L. for 7 days.
- B4: Iron‐sufficient rats orally administered daily for 7 days with 1 mL of the vehicle (distilled water) designated as IS‐control.

#### 2.3.5 Body weight of rats

The initial body weight of all rats was measured and then recorded in the 3rd and 6th week. The body weight of all groups was also determined on the 7th day of the plant dose.

#### 2.3.6 Sample collection of blood and tissues

The rats were anesthetized in chloroform vapor. When they became unconscious, rats were dissected, and blood was collected from the hearts of all rats for hematology and liver function tests. The heart, kidney, and liver were collected and stored in 10% formalin for histopathological studies (Sheth et al., 2021).

#### 2.3.7 Relative organ weight

The absolute organ weight of the liver, heart, and kidney was recorded and the relative organ weight or coefficient of organ weight was calculated according to He et al. (2019).

#### 2.3.8 Hematological analysis

Blood parameters of collected samples that include red blood cells (RBCs), hemoglobin, hematocrit (HCT), MCH, MCV, and MCHC were determined by an automated hematology analyzer.

#### 2.3.9 Liver function test

All blood samples were centrifuged at 3000 rpm for 15 min. Then serum was separated for liver function tests (ALP, SGPT (ALT), SGOT (AST)), and total bilirubin were determined from Sheikh Zaid hospital, Lahore.

#### 2.3.10 Histopathology

For histological studies, liver, kidney, and heart were analyzed and examined. After fixing, the organs were embedded in paraffin, sectioned at 4 mm, stained with hematoxylin and eosin, and examined microscopically by using the procedure of Sheth et al. (2021).

#### 2.3.11 Osmotic fragility test

The osmotic fragility test was carried out by spectrophotometric method according to the method of Barbosa Filho et al. (2014).

#### 2.3.12 Catalase activity

Catalase activity of serum was measured by using the protocol of Lazarte et al. (2015).

### 2.4 GC–MS analysis

The GC–MS analysis of methanolic leaf extracts of *E. sativa* was carried out by using Gas Chromatography‐Mass Spectrometer (Shimadzu QP2010 series, Tokyo, Japan) by following the methodology of Arora et al. (2014).

### 2.5 Network pharmacology profiling

#### 2.5.1 Screening of compounds

Physicochemical properties, 3D structures, and Canonical SMILES of all the extracted compounds through GC–MS analysis were obtained from the PubChem (http://www.pubchem.ncbi.nlm.nih.gov/ accessed on August 15, 2023) and SpiderChem (SpiderChem http://www.chemspider.com/ accessed on August 15, 2023) databases. This information was retrieved using the compound names and CID/SID numbers. The compounds Canonical SMILES were input into software DataWarrior V5.5.0 for an in‐depth analysis of their pharmacokinetic properties, specifically focusing on their ADMET properties and toxicity. The specific ranges for ADMET properties were selected according to Lipinski's rule of five for drug discovery, i.e., Drug Likeness (DL ≥ 0.18), Oral Bioavailability (OB ≥ 30), molecular weight (MW < 500 Da), hydrogen bond donors (H donor < 5), hydrogen bond acceptors (H acceptor < 10), and octanol water coefficient (*p* < 5). The identified novel compounds were further subjected to target screening (Li et al., 2019).

#### 2.5.2 Target screening of active compounds and iron deficiency anemia

The putative targets of the selected active compounds were identified by using SwissTargetPrediction, and STITCH database. Subsequently, the potential targets associated with iron deficiency anemia were retrieved from GeneCards and DisGeNET databases, specifically focusing on data related to the species *Homo sapiens*. To eliminate redundancy in the gene list, duplicate entries were removed. The standard names of the targets were then obtained via UniProtKB (https://www.uniprot.org/ accessed on August 15, 2023). A Venn diagram was created by using bioinformatics tool (https://bioinformatics.psb.ugent.be/webtools/Venn/ accessed on 16 August 2023) to identify common gene targets (Zhang et al., 2021).

#### 2.5.3 Gene ontology and enrichment analysis

The data of gene ontology and KEEG pathway was collected by DAVID database (http://david.ncifcrf.gov/ accessed on 16 August 24, 2023). GO analysis is employed to classify gene functions into biological processes (BP), cellular components (CC), and molecular functions (MF). GO annotation and pathway enrichment analysis were performed on 61 common targets by inputting into DAVID, specifying *Homo sapiens* as the chosen species and the probability score selected for enriched pathways was below 0.05. To visually represent these GO annotations and KEGG pathways, dot plots were generated using Shiny GO V0.77 by selecting top 20 pathways (Lu et al., 2021).

#### 2.5.4 Compound‐target network construction by Cytoscape

The compound‐target network was built by using Cytoscape V3.10.1 (https://cytoscape.org/ accessed on August 27, 2023) between active components of *E. sativa* and the iron deficiency anemia therapeutic target. The network underwent filtration using the “degree” parameter, a network node attribute that quantifies the number of connected nodes associated with a specific network node (Xia et al., 2020).

#### 2.5.5 5 hub genes prediction and construction of protein–protein interaction network

The correlation among the therapeutic targets for iron deficiency anemia was assessed by utilizing the STRING database V12.0 (https://string‐db.org/ accessed on August 30, 2023) and the organism selected was *Homo sapiens*. The minimum required interaction was selected to a threshold of 0.4 or higher. The Protein–Protein Interaction (PPI) network was visualized by using Cytoscape V3.10.1 (https://cytoscape.org/ accessed on August 30, 2023). The CytoHubba plugin was subsequently employed to identify hub genes and nodes to a greater degree, which were then referred to for further investigation. A higher degree of connectivity indicates a stronger association among the targeted genes (Lu et al., 2021).

#### 2.5.6 Target‐compound‐pathway network construction

Based on KEGG pathway analysis, the top 10 pathways were analyzed using DAVID to create the target‐compound‐pathway network with Cytoscape (Tao et al., 2013).

#### 2.5.7 Drug Re‐matching or repurposing

Mechanism of action, target and FDA‐approved drugs of top 10 hub gene was further analyzed from a cutting‐edge drug repurposing library (http://www.broadinstitute.org/ accessed on September 15, 2023) (Rose et al., 1999).

## 3 RESULTS AND DISCUSSION

**FIGURE 1:**
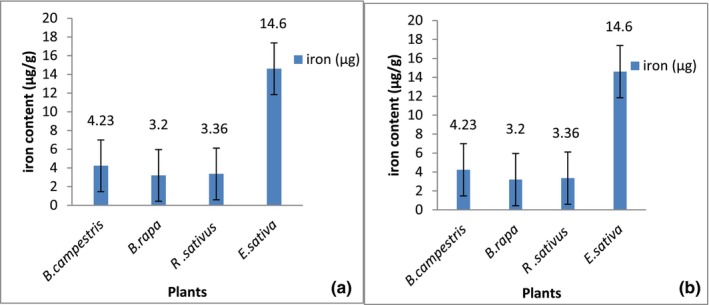
Preliminary in vitro tests. (a) Iron content of selected plants spp. (b) Vitamin C content of selected plants spp.

The current study indicated that *E. sativa* exhibited higher iron (Figure 1) and Vitamin C content among all selected members (Figure 1). Iron and vitamin C have a synergistic relationship when it comes to human nutrition. Vitamin C improves the absorption of non‐heme iron, the type of iron that is found in plant‐based foods and supplements (Hu et al., 2022). It transforms the iron from a less absorbable form to a more soluble form, increasing its uptake in the intestines (Kontoghiorghes et al., 2020).

**FIGURE 2:**
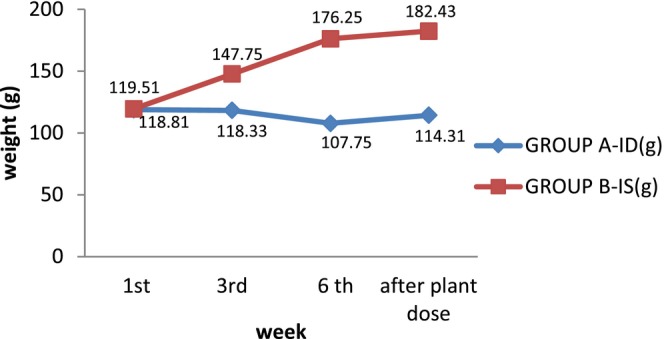
Body weight of rats group A‐ID (iron deficient) and group B‐IS (Iron sufficient).

The average initial weight of all animals was similar. After 6 weeks, the weight of anemic rats of group A (ID) was decreased as compared to non‐anemic group B (IS) (Figure 2). Iron deficiency anemia leads to weight loss due to several factors. Due to less hemoglobin, cells receive less oxygen, leading to fatigue and lessened physical activity, which contributes to weight loss. Anemia also leads to muscle weakness, decreased muscle mass, and reduced appetite and impairs the body's ability to absorb nutrients, leading to decreased food intake and weight loss (Xiao et al., 2016). On the 7th week, administration of extract of *E. sativa* significantly increased (*p* < .01) body weight in both A (ID) and B (IS). (He et al., 2019) also investigated that the body weight of anemic rats reduced (*p* < .05) due to anemia.

**FIGURE 3:**
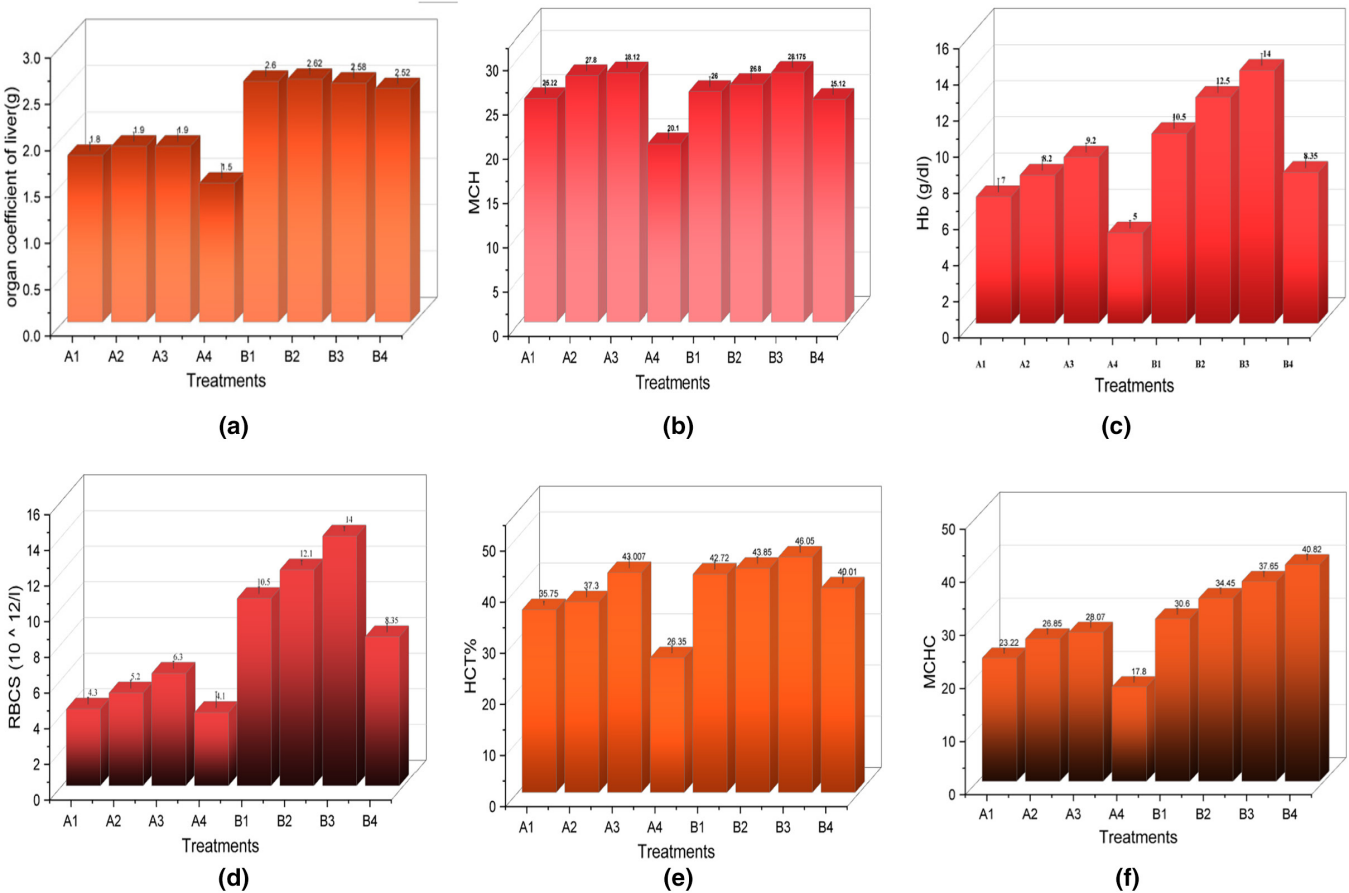
In vivo screening of *E. sativa* extracts. (a) Organ coefficient of the liver. (b) Macrocytic anemia (MCH). (c) Hemoglobin (Hb). (d) Red blood cells (RBCs). (e) Hematocrit test (HCT). (f) Mean corpuscular hemoglobin concentration (MCHC).

The organ coefficient is a crucial aspect of drug safety evaluation, reflecting the extent of internal organ disease. The relative organ weight of the kidney and liver in all groups exhibited no significant change, while the organ coefficient of the liver was decreased in anemic rats in group A4 (ID‐control) as shown in Figure 3a,b. Atrophy of the liver was improved by the oral dosage of *E. sativa*. Iron deficiency leads to tissue hypoxia, including the liver, resulting in a decrease in relative organ weight (Yun et al., 2011).

In the current study, the Hb level was significantly less in group A4 (ID‐control) that was fed an iron‐deficient diet, as compared with group B4 (IS‐control). Lower levels of iron cause less production of hemoglobin and thus lower the oxygen transport in the body (Sun et al., 2022), while Hb content significantly increased (*p* < .0001) in both groups A(ID) and B(IS), which were treated with plant doses of 200,400, and 800 mg/kg, respectively, in a dose‐dependent manner Figure 3c. *E. sativa* is rich in nutrients and phytochemicals, due to the high profiling of iron, folate, and vitamin C; it enhances the Hb level of all groups. The presence of ascorbic acid in *E. sativa* not only enhances Hb levels but also improves iron absorption in the body (Taviano et al., 2017).

In the present study, the HCT, red blood cells, and MCHC were less in the anemic A4 (ID‐control) as compared to B4 (IS‐control). Oral administration of *E. sativa* enhanced the blood parameters in a dose‐dependent manner shown in Figure 3d–f. Suzana et al. (2017) also observed that the HCT% was reduced in the anemic group as compared to the group that was administered by Moringa extract. When iron is deficient in the body, it can lead to a decrease in the production of red blood cells and a decrease in hematocrit levels. This is because, without sufficient iron, the body is unable to produce an adequate amount of hemoglobin, resulting in a lower volume of red blood cells (Salahat & Ibrahim, 2012).

**FIGURE 4:**
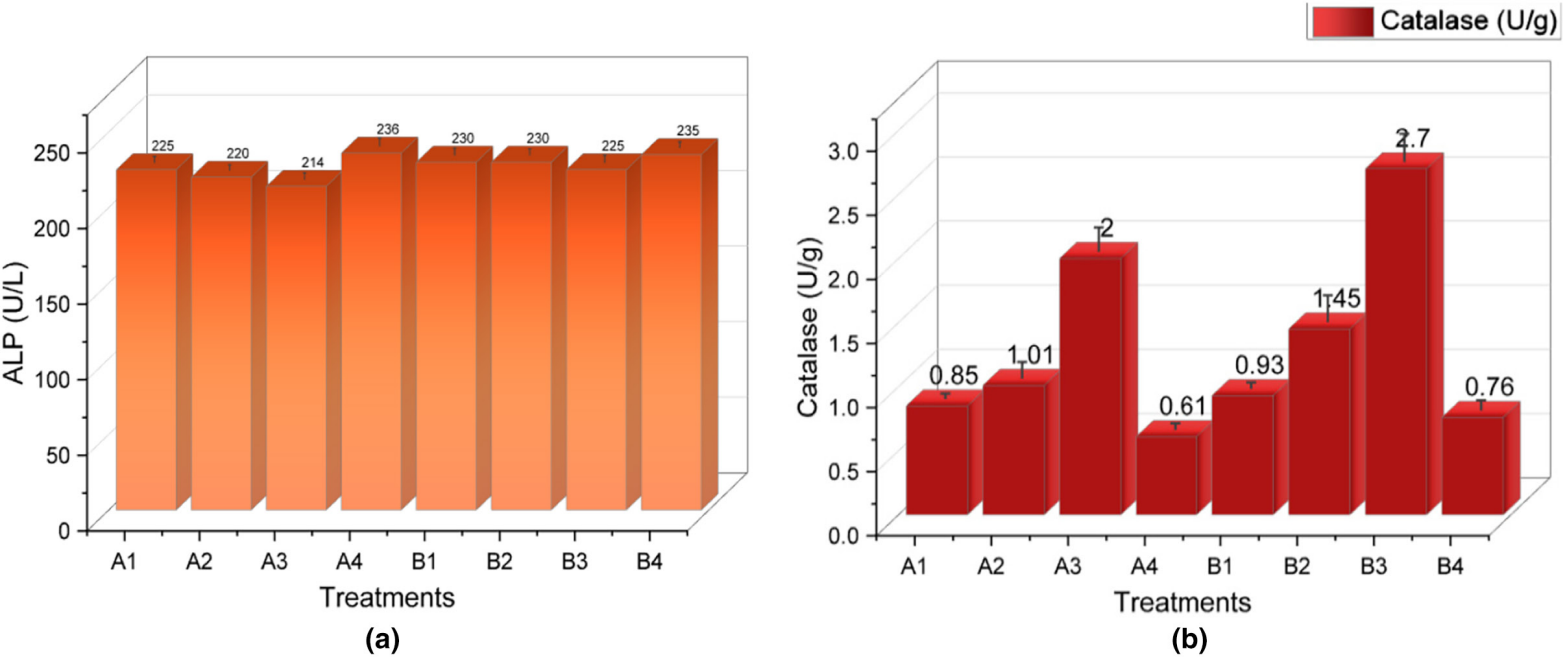
(a) Alkaline phosphatase (U/L) (b) Serum catalase activity (U/g).

Liver function tests typically include measurements of liver enzymes (such as ALP, SGPT SGOT, and total bilirubin levels). In the present study, alkaline phosphatase (ALP) was affected in group A4(ID‐control) due to deficiency anemia shown in Figure 4a. Its concentration was improved in the group that was given by plant extract. Iron deficiency anemia leads to a decrease in the production of red blood cells, resulting in reduced oxygen‐carrying capacity. Iron deficiency leads to tissue hypoxia and inadequate oxygenation of various organs, including the liver. In response to this hypoxia, the liver releases an enzyme called alkaline phosphatase. Therefore, an increase in alkaline phosphatase levels is observed in individuals with iron deficiency anemia as a compensatory mechanism to improve the oxygenation of tissues (Shi et al., 2023).

**FIGURE 5:**
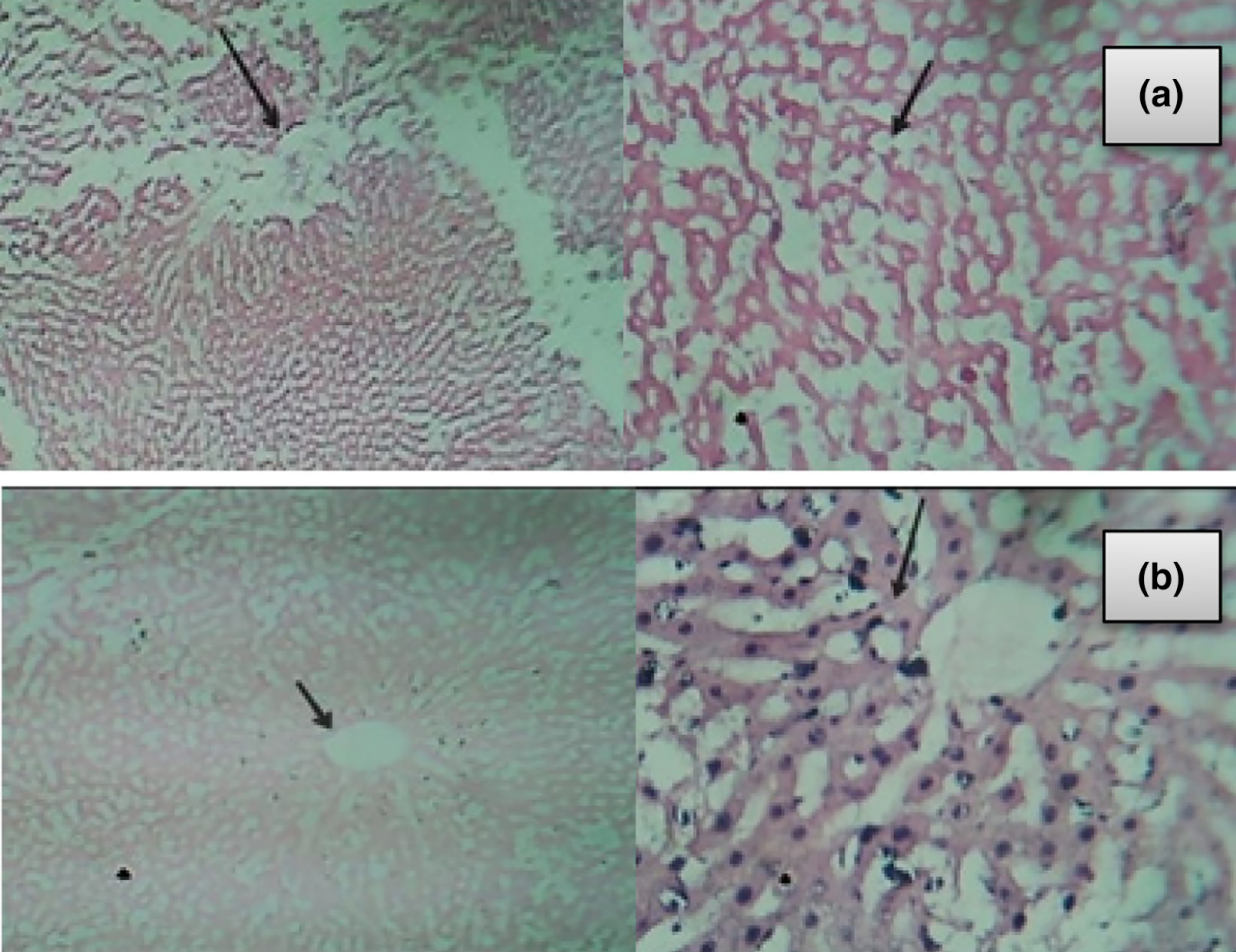
Histopathology of liver. Micrograph of (a) anemic liver and (b) non‐anemic liver.

In the present study, in anemic liver hepatocytes were not cleared with disorganized dilated sinusoids, and tissues were damaged (Figure 5). The liver plays a vital role in iron absorption and iron metabolism. Due to iron deficiency anemia, various changes occur in the liver. Low iron concentration affects the hepcidin, which leads to certain changes in the liver (Sun et al., 2022), while the oral supplementation of *E. sativa* meliorated the effect of iron deficiency. (Sun et al., 2022) also declared that due to iron deficiency, hepatocytes were damaged and that effect was reduced by *Tegillarca granosa* extract (Figure 7).

**FIGURE 6:**
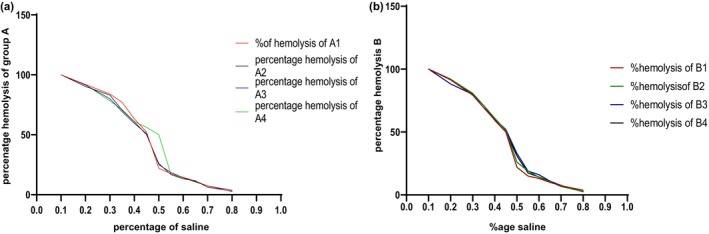
Osmotic fragility curve of (a) group A (ID) and (b) group B(IS).

**FIGURE 7:**
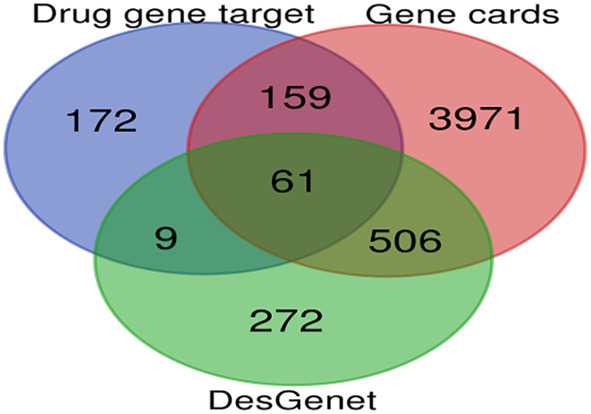
Venn diagram showing common targets.

**FIGURE 8:**
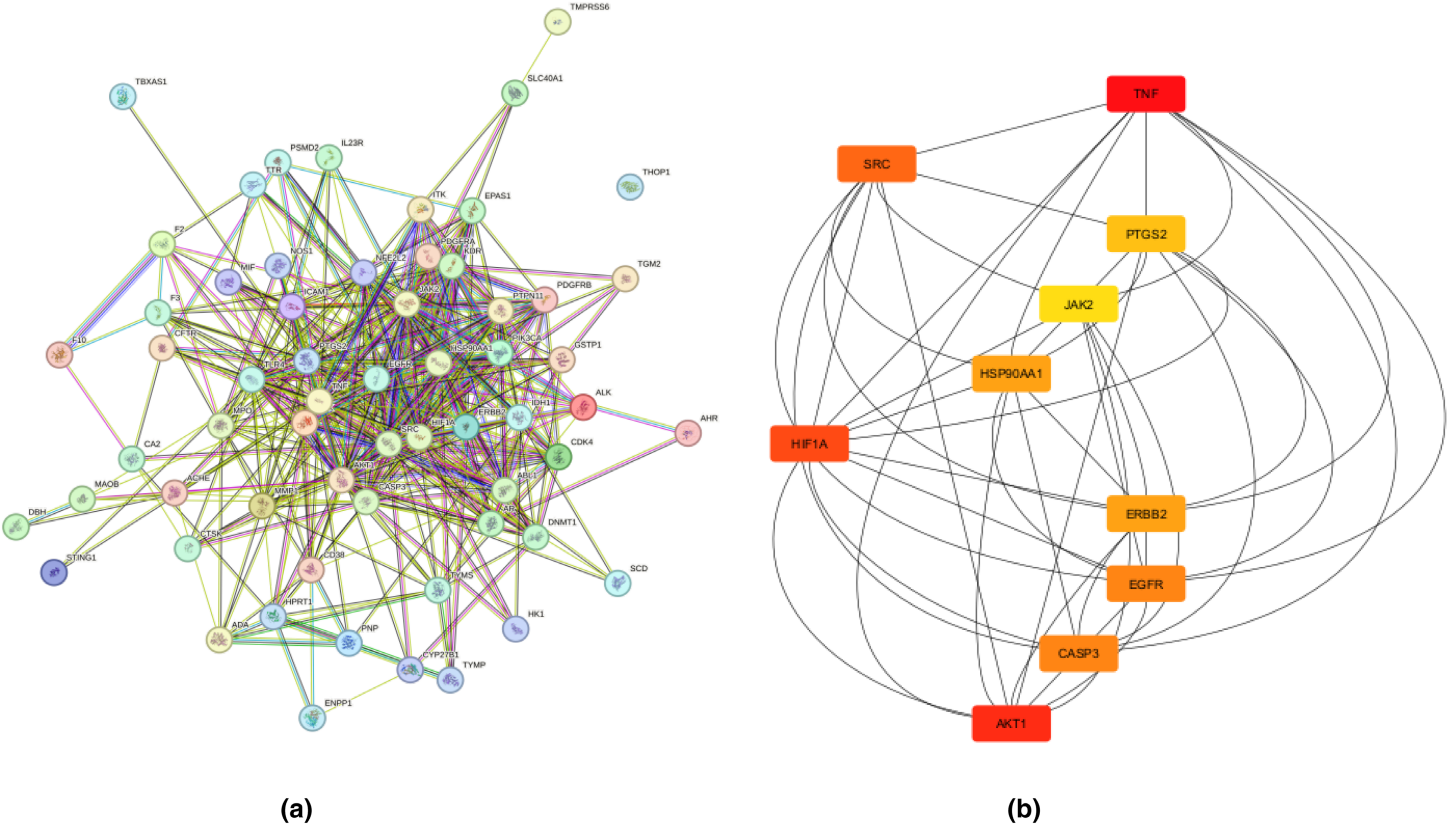
(a) Protein–Protein interaction network. (b) Hub genes.

In recent studies, the osmotic fragility of anemic rats was increased due to iron deficiency (Figure 6a), while the osmotic fragility of B(IS) was not altered (Figure 6b). Administration of *E. sativa* extract significantly (*p* < .01) improved the osmotic fragility of red blood cells. Iron deficiency makes red blood cells more susceptible to swelling and lysis when exposed to a hypotonic solution (Figure 8a and 8b). The lower concentration of hemoglobin in the cells also contributes to their increased fragility (Öztürk et al., 2017).

In the present study, catalase activity of serum in anemic rats A4 (ID control) was lowered due to oxidative stress (Figure 4b), while catalase activity was enhanced (*p* < .001) in the groups that were given *E. sativa* doses in both groups A(ID) and B(IS). *E. sativa* is rich in antioxidants and ascorbic acid that stimulate enzyme activity and detoxify reacting oxygen species (Keyata et al., 2020). Parvaz et al. (2022) reported that iron deficiency causes a reduction in the activity of the catalase enzyme. It was observed that CAT activity was significantly increased (*p* < .05) in the group that was administered by *Pistacia vera* extract.

Discovery and the development of novel drugs are expensive and time‐consuming processes. A planned computational approach for rational drug design has been useful in assessing the potential of selected phytochemicals (Khan et al., 2018). The GC–MS analysis of methanolic leaves extract of *E. sativa* revealed 79 compounds. After performing ADMET analysis on these 79 compounds, 6 compounds – glucocochlearin, glucoiberverin, glucoerucin, 4‐mercaptobutyl glucosinolate, 4‐(methylthio) butyl thiocyanate, and bis(4‐isothiocyanatobutyl) disulfide – were selected as effective compounds by applying Lipinski's rule of five. Based on this rule, these six compounds conform to the criteria without any violations (Table 2). The intricacies of drug development stemming from natural resources result in methodological challenges. The recently created medication is limited in its effectiveness due to the absence of ADME properties, and the cost‐intensive nature of development adds further hurdles to drug discovery approaches. As a result, pharmaceutical professionals place significant emphasis on in silico ADME‐based screening methods in the field of medication development (Bocci et al., 2017) (Mukhtar & Khan, 2023).

**TABLE 2:** Physicochemical, pharmacokinetics properties and 3D structure of *E. sativa.*

Sr. no.	Phytochemicals	3D structure	Molecular weight (MW < 500 Da)	Clog P	H‐bond acceptors (<10)	H‐bond donor (<5)	Drug likeness (DL ≥ 0.18)	Oral bioavailability (OB ≥ 30)	Polar surface area	No. of rotatable bonds (<10)
1	Glucocochlearin	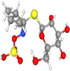	375.418	−1.0641	10	5	−0.52781	0.11	199.79	7
2	Glucoiberverin	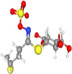	406.476	−2.3808	10	4	−6.0824	0.11	227.92	9
3	Glucoerucin	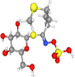	421.511	−0.6788	10	5	−3.6436	0.11	225.09	10
4	4‐Mercaptobutyl glucosinolate	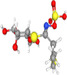	407.484	−0.8952	10	5	−13.434	0.11	238.59	9
5	Bis(4‐isothiocyanatobutyl) disulfide	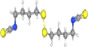	292.515	6.2504	2	0	−5.4271	0.55	139.5	11
6	4‐(Methylthio)butyl thiocyanate	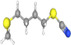	161.292	1.9154	1	0	−6.4689	0.55	74.39	5

Efficacy and toxicity are important factors in drug discovery. Rapid in silico process is the best way to predict the toxicity of active compounds derived from plants; this reported information will be helpful in novel drug formulation with fewer side effects (Husain et al., 2022). The toxicity assessment of identified compounds revealed that glucocochlearin, glucoiberverin, glucoerucin, and 4‐mercaptobutyl glucosinolate have no mutagenic, tumorigenic, or irritant effects, whereas bis(4‐isothiocyanatobutyl) disulfide and 4‐(methylthio)butyl thiocyanate have mild toxic effect mutagenic, tumorigenic, and irritant. Moreover, all the compounds except glucoerucin appeared to have mild to slightly high reproductive toxicity effects (Table 3).

**TABLE 3:** Prediction of toxicity risk assessment of active compounds of *E. sativa.*

Phytochemicals	Mutagenic	Tumorigenic	Reproductive	Irritant
Glucocochlearin				
Glucoiberverin				
Glucoerucin				
4‐Mercaptobutyl glucosinolate				
Bis(4‐isothiocyanatobutyl) disulfide				
4‐(Methylthio)butyl thiocyanate				

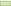
, non‐toxic; 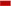, highly toxic; 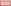, low toxic.

**FIGURE 9:**
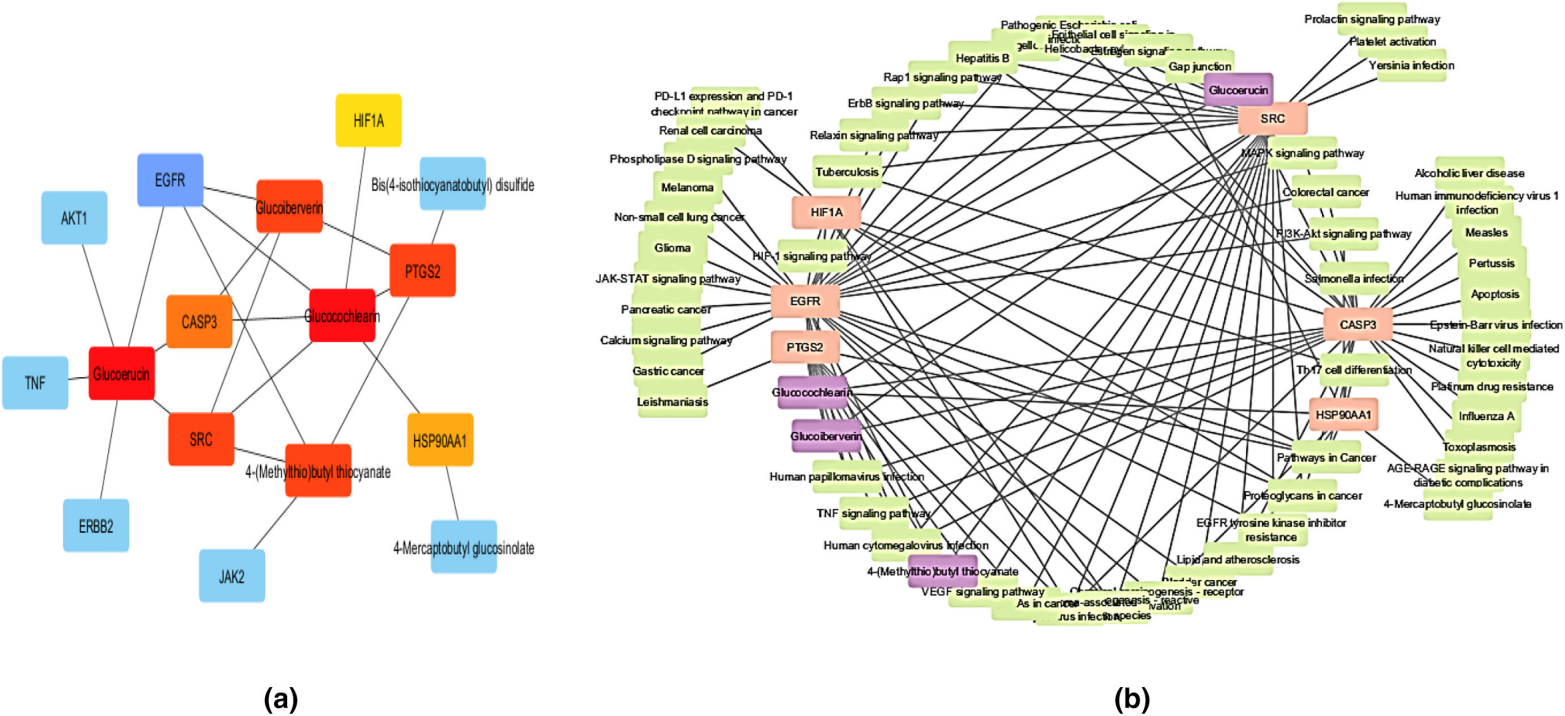
(a) First‐stage nodes genes by cytoHubba. (b) Compound‐Target network.

A total of 745 targets were retrieved from 6 active chemical constituents of *E. sativa* through SwissTargetPrediction. The potential targets of iron deficiency anemia were found in the GeneCard and DisGeNet databases, which yielded 4677 and 848 results, respectively. After removing duplication and potential mapping of drug and disease targets, a total of 61 common targets were identified (Figure 9). These targets were then considered as potential targets against iron deficiency anemia.

**FIGURE 10:**
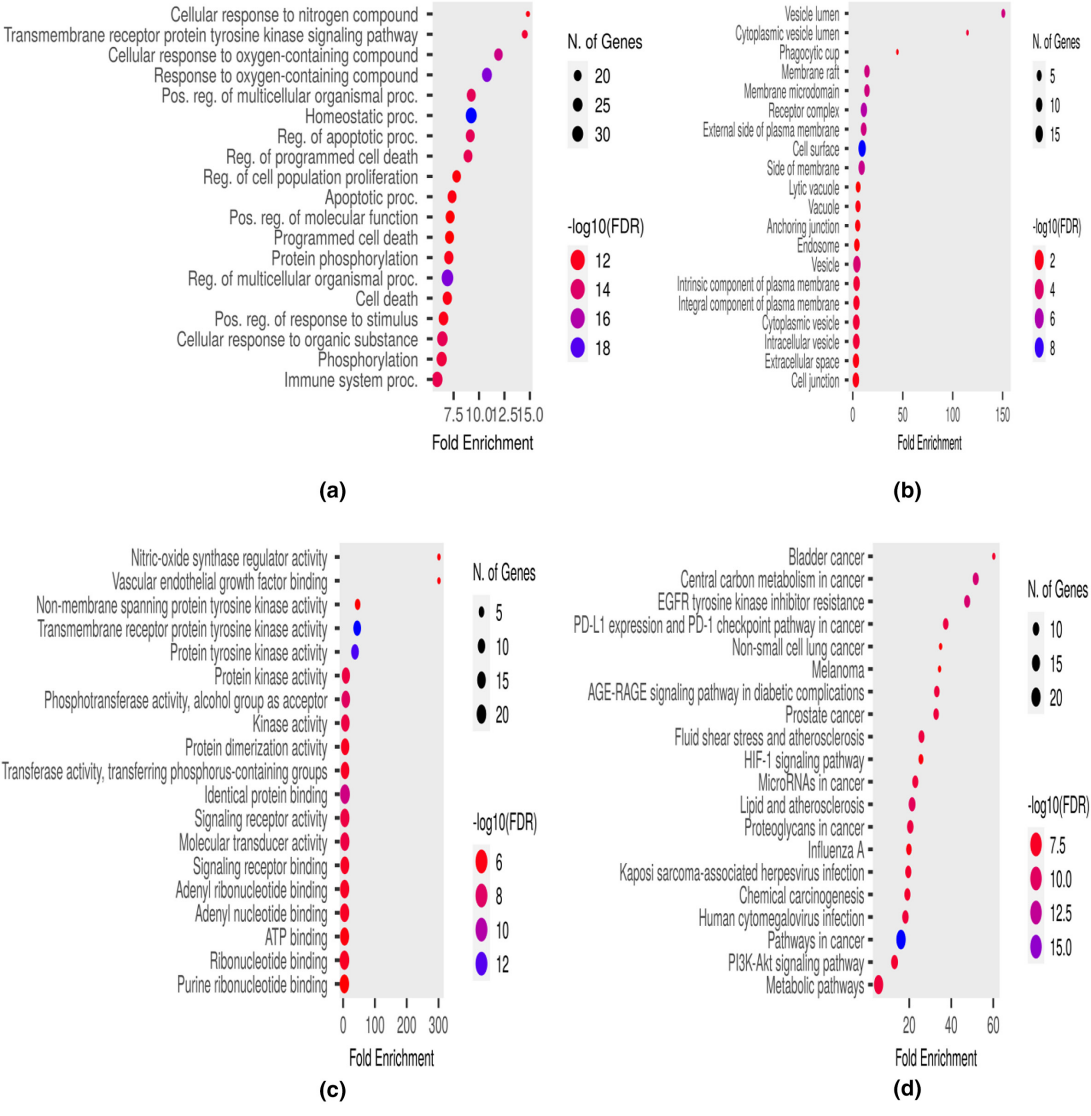
Gene ontology and enriched pathway analysis. (a) GO Biological process (BP). (b) GO Cellular component. (cc) (c) GO Molecular function (MF). (d) KEEG Pathway.

Protein–protein interaction was built between 61 common genes through STRING database. Protein–protein interactions play a pivotal role due to their remarkable versatility, adaptability, and selectivity. Analyzing protein–protein interactions unveils the connections and correlations among the molecular targets (Cai et al., 2019). The network was visualized in Cytoscape, 61 nodes with 435 edges were found. In Figure 10, network nodes represent the proteins, i.e., colored nodes: red color nodes representing query proteins and the first shell of interactions and white color nodes representing the second shell of interactions; content nodes: empty nodes representing proteins of unknown 3D structure and filled nodes representing a 3D structure of known and predicted. Edges represent protein–protein interaction i.e. sky blue (from curated databases) and purple line (experimentally determined) interactions representing known interaction; green (gene neighborhood), red (gene fusion), and blue (genes co‐occurrence) lines representing predicted interactions; and parrot, black, and light blue representing the other interactions like text mining, co‐expression, and protein homology, respectively (Figure 8a).

Then, cytoHubba plugin was used to find out the hub genes by using degree methods and selecting top 10 nodes. The highest degree shows more connectivity of the targets with other ones, and it is also an indication of key targets. The top 10 genes identified were TNF (45), AKT1 (38), HIF1A (35), SRC (34), EGFR (33), CASP3 (33), HSP90AA1 (31), ERBB2 (31), PTGS2 (29), and JAK2 (28) (Figure 8b, Table 4). Zimmermann et al. (2006) and Lamb et al. (2006) also identified TNF, EGFR, and AKT1 anemia‐related targets.

**TABLE 4:** Top 10 hub genes identification by degree method in cytoHubba.

Rank	Genes	Degree score
1	TNF	45
2	AKT1	38
3	HIF1A	35
4	SRC	34
5	EGFR	33
6	CASP3	33
7	HSP90AA1	31
8	ERBB2	31
9	PTGS2	29
10	JAK2	28

**FIGURE 11:**
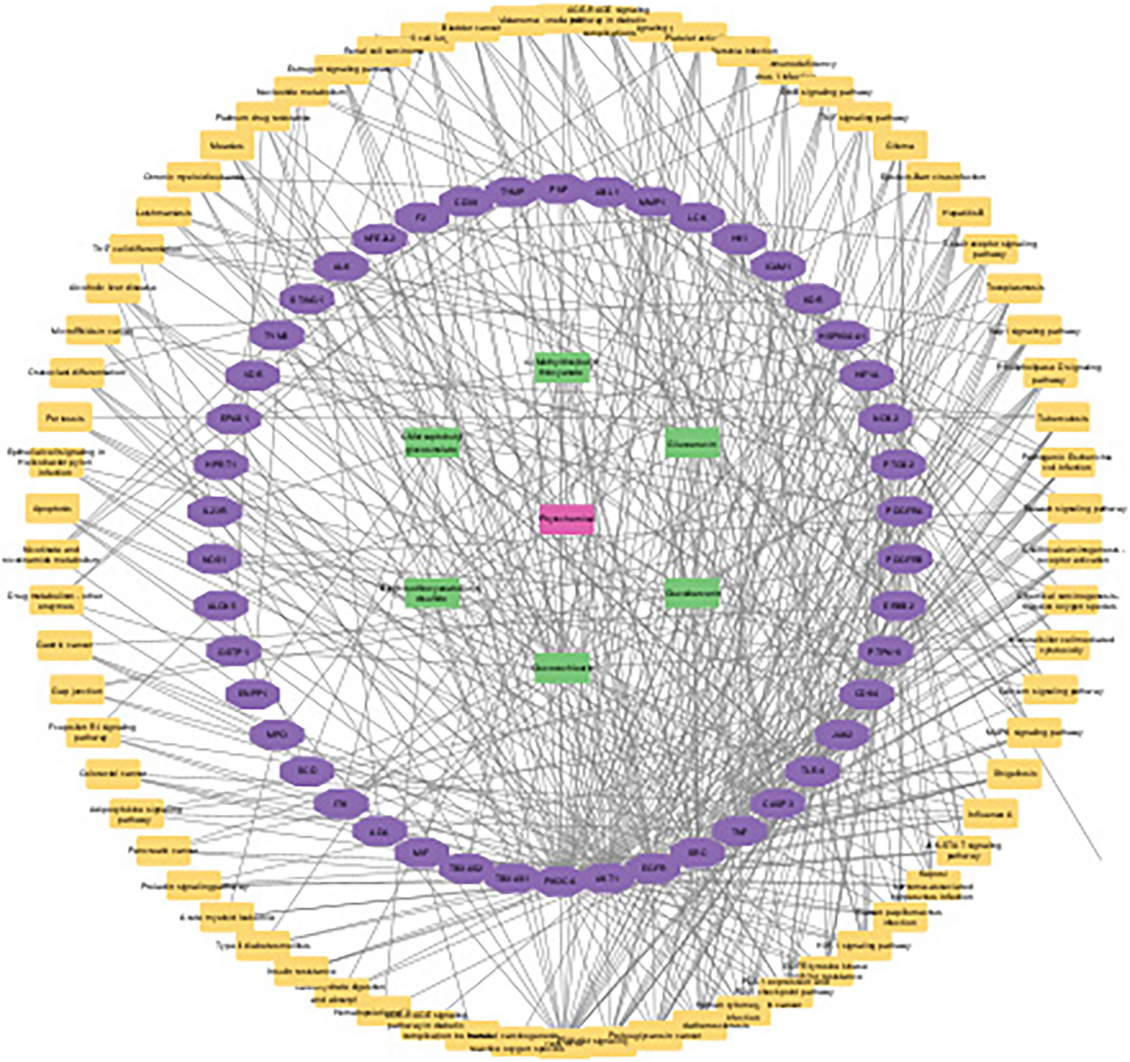
Compound‐target‐pathway network.

Further study was performed on the molecular mechanism of *E. sativa* targets in treatment of iron deficiency anemia. For this purpose, GO analysis was performed to identify the gene function at molecular, cellular, and biological levels. After applying cutoff value *p* < .05, GO analysis generated 197 biological processes which included regulation of auto‐phosphorylation, regulation of biosynthetic process, regulation of cell and fibroblast proliferation, regulation of growth factors and signaling pathways, regulation of gene expression, cell response to drug, xenobiotic metabolic process, morphogenesis of an epithelial fold, proteolysis, and metabolic processes (Figure 10a); 31 cellular components which included macromolecular complexes, receptor complexes, plasma membrane, cytoplasm, and nucleoplasm (Figure 10b); and 32 molecular functions which included extracellular regions, serine‐type endopeptidase complex, intrinsic component of plasma membrane, nucleus, neuronal cell body, receptor complex, and transcription factor complex (Figure 10c). Same observation was made by Lihong et al. (2023) and Wang et al. (2023) in these iron deficiency anemia‐related targeted pathway. After that 20 Go annotations (BP, CC, and MF) and KEGG pathways were selected to draw the dot plot for visual representation (Figure 10d). A compound‐pathway network was also built to show the KEGG pathways involved in this study (Figure 11).

A network between potential targets and active compounds was constructed using Cytoscape to analyze the interaction between the active compounds and potential targets. A compound‐target network was created with potential top hub target and four active ingredients of *E. sativa*. Analyzing the network with a “network analyzer” shows that the compound‐target network comprises 65 nodes and 115 edges.

The mechanism of *E. sativa* in iron deficiency anemia was studied using network analysis. For this, top enriched pathways were selected by DAVID analysis, and the target–pathway–compound network was created with Cytoscape. There were 102 nodes and 311 edges in the network, with 6 active components, 61 potential targets, and relevant pathways (Figure 9a,b). The targets of *E. sativa* active components show coordination with diverse paths and are connected to each other and play a major role in the treatment of Iron deficiency, which widely embodies traditional Chinese medicine's multi‐target, multi‐component, and multi‐pathway physiognomies.

**FIGURE 12:**
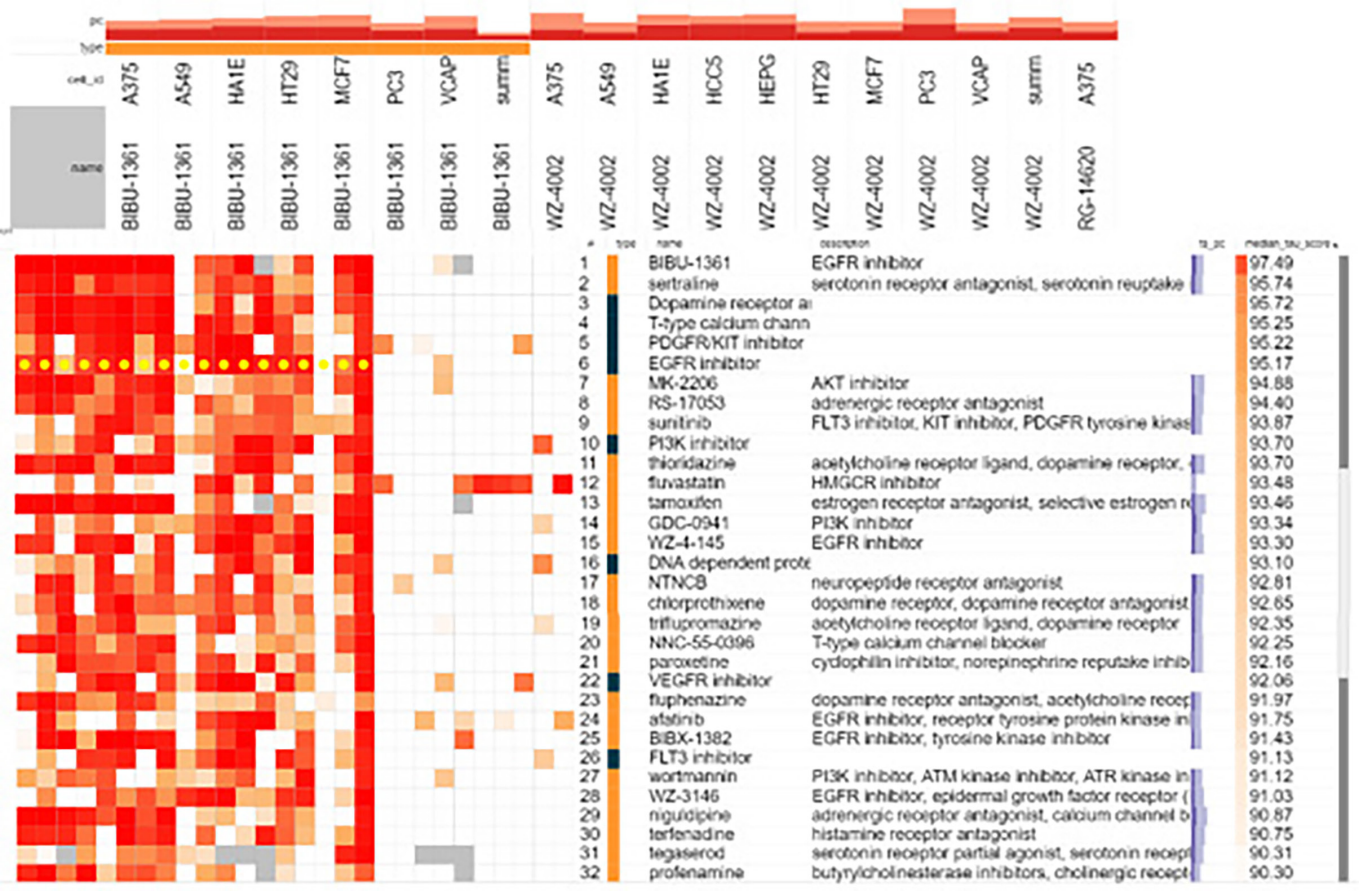
Small molecule drug associated with EFGR target analyzed and screened from drug repurposing hub (Median score ≥90).

Online drug repurposing hub (http://www.broadinstitute.org/) database was used to analyze the relationship between top hub gene EGFR and related drug targets (Table 5). The epidermal growth factor receptor (EGFR) belongs to ErbB family, which also includes HER‐2, HER‐3, and HER‐4. Natural ligands, primarily EGF and transforming growth factor alpha (TGF‐), activate the receptor, promoting activation of the intracellular tyrosine kinase, which inhibits apoptosis, cell proliferation, and angiogenesis (Table 5, Figure 12).

**TABLE 5:** Top drug‐targeting EFGR gene and their mechanism of action.

Drug ID	Name	Description	Target	MOA
BRD‐K13087974	4,5‐dianilinophthalimide	EGFR inhibitor	EGFR	EGFR inhibitor
BRD‐K68407802	KIN001‐055	EGFR inhibitor	EGFR, JAK3	EGFR inhibitor, JAK inhibitor, Leukotriene inhibitor, Mediator release inhibitor
BRD‐K64052750	Gefitinib	EGFR inhibitor	EGFR, CYP2C19	EGFR inhibitor
BRD‐K72420232	WZ‐4002	EGFR inhibitor	EGFR, ERBB2	EGFR inhibitor
BRD‐K70914287	BIBX‐1382	EGFR inhibitor	EGFR, ERBB2	EGFR inhibitor, tyrosine kinase inhibitor
BRD‐U25771771	WZ‐4‐145	EGFR inhibitor	CSF1R, DDR1, EGFR, PDGFRA, TIE1	EGFR inhibitor
BRD‐K32292990	CGP‐53353	EGFR inhibitor	EGFR, PRKCB	EGFR inhibitor, PKC inhibitor
BRD‐K50168500	Canertinib	EGFR inhibitor	EGFR, ERBB2, ERBB4, AKT1	EGFR inhibitor
BRD‐K66175015	Afatinib	EGFR inhibitor	EGFR, ERBB2, ERBB4	EGFR inhibitor
BRD‐K68336408	tyrphostin‐AG‐1478	EGFR inhibitor	EGFR, MAPK14	EGFR inhibitor
BRD‐K21853356	RG‐14620	EGFR inhibitor	EGFR	EGFR inhibitor
BRD‐K49294207	BIBU‐1361	EGFR inhibitor	EGFR	EGFR inhibitor
BRD‐K52850071	JAK3‐Inhibitor‐II	JAK inhibitor	EGFR, ALK, JAK1, JAK2, JAK3	JAK inhibitor
BRD‐K73293050	WZ‐3146	EGFR inhibitor	EGFR	EGFR inhibitor

The price of formulating, approving, and purchasing new medications is increasing rapidly. Conversely, sluggish economies find it difficult to fund their healthcare systems, especially in nations where the elderly require more subsidies. Action must be taken right away to solve this problem, and one solution is to research the vast, already‐approved drug supply and apply it for novel cures. In the present study, we have explored the phytochemicals enriched in *E. sativa* leaves. The network pharmacological profiling showed EFGR among the top hub genes.

Due to drug resistance, reusing therapeutic medicines that have failed is typically not advised. In relation to chemotherapy and targeted drugs, clinical evidence over the past few decades has revealed a role for unstable, non‐heritable pathways of acquired drug resistance (Kuczynski et al., 2013). Pharmacological perturbations by synthetic chemicals or natural compounds activate cellular signal transduction and generate particular gene expression patterns after binding to the drug targets (Lamb et al., 2006). Radiation therapy (RT) can cause anemia or exacerbate anemia that already exists, and this impact is amplified if concurrent systemic therapy is given (Harrison et al., 2001). EGFR is overexpressed in 80%–90% of head and neck cancer cases, and they are linked to worse rates of loco‐regional control and survival after radiotherapy (RT). Interleukin 6 (IL‐6) production is elevated because of EGFR activation, and RT's inflammatory response can intensify this effect. Hepcidin, a crucial protein in the control of iron metabolism, is produced in greater amounts as a result of IL‐6 (Zimmermann et al., 2006). Hepcidin increases the amount of iron that is trapped in the liver, preventing it from reaching hematological cells and causing a functional iron deficit, which may account for the increased anemia rates observed in RT patients. Cell toxicity results from excessive body iron concentrations brought on by low hepcidin levels. Oxidative stress and the production of reactive oxygen species are caused by high iron levels in the cells. The heart, liver, and hormone glands may become dysfunctional as a result (Fung & Nemeth, 2013). Interleukin 6 (IL6) production is upregulated in response to epidermal growth factor receptor (EGFR) activation, which is exacerbated by the inflammatory response brought on by radiation (RT). EGFR inhibitor cetuximab has been linked to lower anemia rates in patients with locally advanced head and neck squamous cell carcinoma (HNSCC) (Grellier et al., 2015). Although cetuximab provides survival benefits when combined with chemotherapy, cutaneous toxicities related to cetuximab affect the quality of life of patients. Based on drug repurposing and network pharmacological assessment, the present study suggested that screened compounds can be used in discovering novel indications and repurpose existing EFGR inhibitor‐resistant drugs (Bou‐Assaly & Mukherji, 2010).

## 4 CONCLUSION

The research findings provide compelling evidence regarding the promising therapeutic implications of utilizing the aqueous extract derived from *Eruca sativa* (L.) as a treatment modality for iron‐deficient anemia. This plant exhibits a noteworthy capacity to enhance various critical health indicators in iron‐deficient rats. Notably, it contributes to the augmentation of hematological parameters, antioxidant activity, enhancing liver function, and fortifying erythrocyte stability. These observed positive outcomes can be attributed to the presence of bioactive constituents within the extract, which are believed to play a pivotal role in driving these beneficial effects. Network pharmacology analysis revealed these compounds are also involved in regulation of various gene functions at molecular, cellular, and biological levels. Furthermore, a noteworthy observation is the dose‐dependent relationship between the extract and the improvement in all examined parameters related to blood and liver. This suggests a potential avenue for tailored treatments based on extract dosage, opening new possibilities for the management of iron‐deficient anemia.

## AUTHOR CONTRIBUTIONS

**Sana Javed:** Investigation (equal); writing – original draft (equal). **Zainab Shahzadi:** Investigation (equal); writing – original draft (equal). **Zubaida Yousaf:** Conceptualization (equal); project administration (equal); supervision (equal); validation (equal). **Irfan Anjum:** Project administration (equal); supervision (equal). **Arusa Aftab:** Methodology (equal); software (equal). **Samina Hanif:** Formal analysis (equal); software (equal). **Riaz Ullah:** Funding acquisition (equal); resources (equal); supervision (equal); writing – review and editing (equal). **Muhammad Ahmer Raza:** Validation (equal); visualization (equal). **Zafar Iqbal:** Software (equal); writing – review and editing (lead).

## FUNDING INFORMATION

The authors wish to thank Researchers Supporting Project Number (RSPD2024R706) at King Saud University Riyadh Saudi Arabia for financial support.

## CONFLICT OF INTEREST STATEMENT

The authors have no conflict of interest.

## ETHICS STATEMENT

Before starting the experiment, the study was approved by the institutional research Ethics Committee of the Faculty of Pharmacy, The University of Lahore, and research Ethical Committee of Lahore College for Women University, Lahore, Pakistan (Ref. no. Bot/LCWU/1521‐A).

## Data Availability

The data will be available upon reasonable request made to the corresponding author.
